# PROTOCOL: Megamap of systematic reviews and evidence and gap maps on the effectiveness of interventions to improve child well‐being in low‐ and middle‐income countries

**DOI:** 10.1002/cl2.1057

**Published:** 2019-10-10

**Authors:** Ashrita Saran, Kerry Albright, Jill Adona, Howard White

**Affiliations:** ^1^ Campbell Collaboration Delhi India; ^2^ UNICEF Office of Research‐Innocenti Florence Italy; ^3^ Philippines Institute of Development Studies Manila Philippines

## 1 BACKGROUND: THE STATE OF CHILD WELL‐BEING IN LOW‐ AND MIDDLE‐INCOME COUNTRIES

Child well‐being is a multidimensional and a holistic concept which provides a contextual understanding of a child in different domains such as health, material well‐being, education, conditions of housing and environment, and interpersonal relations (UNICEF, 2014). A decent level of child well‐being is underpinned by the Declaration of the Rights of the Child which states that “The child, by reason of his physical and mental immaturity, needs special safeguards and care, including appropriate legal protection, before as well as after birth” (Cohen, 1989). But many children around the world still suffer deficiencies in many dimensions of well‐being.

### 1.1 Aspects of shortfalls in child well‐being

One in three children (200 million globally) fails to reach their full physical, cognitive, psychological and/or socioemotional potential due to poverty, poor health and nutrition, insufficient care, stimulation and other risk factors to early childhood development (Grantham‐McGregor et al., 2007). It is estimated that globally, almost 385 million children are living in extreme poverty. Poverty, malnutrition, poor health, unstimulating home environments and violence against children are major risk factors, which detrimentally affect the cognitive, motor and social‐emotional development of children (Tran, Luchters & Fisher, 2017). Preterm birth complications, acute respiratory infections, intrapartum‐related complications, congenital anomalies and diarrhoea are the main factors continuing causes of high numbers of under‐five deaths (WHO, 2019).

Further adding to the plight of children in developing countries, young girls and adolescent women are invariably subjected to various forms of harmful practices including child marriage and female genital mutilation. Close to 300 million (3 in 4) children aged 2 to 4 worldwide experience violent discipline by their caregivers on a regular basis and approximately 250 million (around 6 in 10) are punished by physical means. Recent estimates of ILO show that Asia and the Pacific still has the largest numbers of child labour (almost 78 million or 9.3% of child population), but sub‐Saharan Africa continues to be the region with the highest incidence of child labour (59 million children which is over 21% of all children; ILO, 2013). In 2017, four African nations (Mali, Benin, Chad and Guinea‐Bissau) witnessed over 50% of children aged 5–14 working (UNICEF, 2017). The worst form of child labour lead to severe exploitation of children including impact on physical, mental and moral and social development of children (ILO, 2013).

Education offers children a path to a promising future but about 264 million children and adolescents around the world fail to enter or complete school. They are thwarted by poverty, discrimination, armed conflict, emergencies and the effects of climate change (UNICEF, 1998) Often the family and environmental risk factors a child experiences are beyond the control. Though the effects of these factors can be moderated and this is where opportunities to promote children's positive mental health and wellbeing lie.

### 1.2 Consequences of shortfalls in child well‐being

Childhood deprivation cannot just take childhood from children, but also have long‐run consequences. Child undernutrition is associated with shorter adult height, less schooling, reduced economic productivity and lower offspring birthweight for women (Victora et al., 2008). Lack of education is a major factor in households remaining poor (Baulch, 2011). Early marriage is bad for the health of the mother – with greater risk of dying during childbirth – and her offspring who are at greater risk of having low birth weight and of dying prematurely (Nour, 2006).

### 1.3 Addressing shortfalls in child well‐being

The provision of services on health, education and safety to all children in the world irrespective of cast, creed, colour and ethnicity is a fundamental right enshrined in the UN Convention on the Rights of the Child.

In recognition to the 2030 Agenda for Sustainable Development, children's rights and well‐being are acknowledged as important for long‐term sustainable development of children. Some sustainable development goals (SDGs) are important reference points for the design of national development strategies for child well‐being such as: end poverty (SDG1), end hunger, achieve food security and improved nutrition and promote sustainable agriculture (SDG2), health (SDG 3), quality education (SDG4), reduce inequality between and within countries (SDG10).

Despite this apparent focus on child well‐being and various international organisations working toward a common goal, striking gaps remains in achieving SDG indicators – as outlined above. Research can play a crucial role in helping to close the remaining gaps in global evidence base for effective interventions. SDG 17 targets 17.16 and 17.18 emphasises increased need for investment in generating sound evidence to improve child well‐being interventions strategies. Though child well‐being interventions have been in use for decades; however, evidence for the effectiveness of these interventions are often scattered, their value is possibly underestimated and their inclusion in national strategies and programmes is rare.

Failure to effectively implement evidence informed interventions represents a key obstacle in the progress of child well‐being system in many low and middle income countries (LMICs) toward achieving the United Nations SDGs. This is partly due to a weak and under‐utilised evidence base that does not give policy makers and programme managers the information needed to make decisions. Both international and national organisations should work together to fill the gaps in evidence and to gain a better understanding of what works and what doesn't in child well‐being.

### 1.4 Why it is important to develop the Megamap

Evidence‐based research and multi‐country experiences make a strong rationale for investing in child well‐being programmes. While evidence‐based policy making is of increasing importance, many agencies commission systematic reviews to inform policy, but due to lack of a central repository, systematic reviews are often duplicated and may give misleading findings if not undertaken to proper standards. Also, the existing evidence base around child well‐being has major gaps. Even the existing research is rarely accessed or used for policy or funding decisions as studies are often scattered across different databases and website.

Evidence maps are an approach to providing an overview of the available evidence, with various approaches adopted to evidence mapping by different agencies over the years (Saran & White, 2018). There has been a rapid growth in evidence and gap maps (EGMs) in recent years, notably in international development (Phillips et al., 2017). A typical EGM provides an overview of primary studies and systematic reviews in a particular policy domain. Since this map has such a broad scope – all of child well‐being – we label it as a Megamap and map only systematic reviews and EGMs.

Based on a systematic search, the proposed Megamap will provide an overview of the evidence of the effectiveness of interventions aimed at improving child well‐being in LMICs using an intervention‐outcome framework. It will identify areas in which there are good bodies of synthesised knowledge to inform policy, and those areas in which there is little or no evidence synthesis. The map will contain effectiveness studies. It does not include other research on child well‐being, including qualitative studies.

The map will inform the identification of priority areas where evidence is currently lacking, such as rigorous systematic reviews of the effectiveness of early marriage interventions, child labour or those in conflict‐affected situations. This will help create a central repository of all the available resource on the effectiveness of child well‐being.

UNICEF Innocenti centre with Campbell Collaboration has published a set of five research briefs highlighting main findings of a preliminary version of the Megamap. As a next step, the two organisations are now working on an evidence and gap on child violence in LMICs – identified in the draft Megamap as an area deficient in evidence synthesis.

### 1.5 Scope of the Megamap

The Megamap will include existing EGMs and systematic reviews which synthesise evidence of the effectiveness of child well‐being interventions in improving child well‐being. The map will be presented in two dimensions: the rows list interventions and sub‐categories, and the columns the outcome domains. Each cell shows systematic reviews and EGMs which contain evidence on that combination of intervention and outcome. Included systematic reviews and EGMs are coded for additional characteristics which can be used in filters, such as country, region and child categories.

We started developing draft the framework by reviewing UNICEF strategy documents, key documents by major funders as WHO, UNDP, DFID, Save the Children and World Bank to name a few. In particular we referred to UNICEF strategic plan 2018–2021: Executive summary (UNICEF Research Brief, 2018) and Global Strategy for Women's and Children's health (Ki‐Moon, 2010). Various stakeholder consultations were carried out to refine the framework.

The final framework aimed to provide an overview of existing systematic reviews for child welfare interventions as

1. Early childhood interventions that addressed the period from pregnancy, child birth and children up to 3 years of age.
2. Health and nutrition that addressed maternal health interventions, timing and spacing of birth, child birth, nutrition, prevention and treatment of childhood diseases.
3. Educational intervention that aimed to address improve learning and achievement and skill development of children from 4 to 18 years of age.
4. Social work and welfare that aimed to protect the child from violence and other risk factors.
5. Social protections interventions that aimed to provide financial support to mother, children and families to access the basic amenities for survival and living.
6. Environmental WASH to ensure every child lives in clean environment.
7. Governance and advocacy to ensure sustainable intervention strategies.

### 1.6 Conceptual framework of the EGM

**Figure 1:**
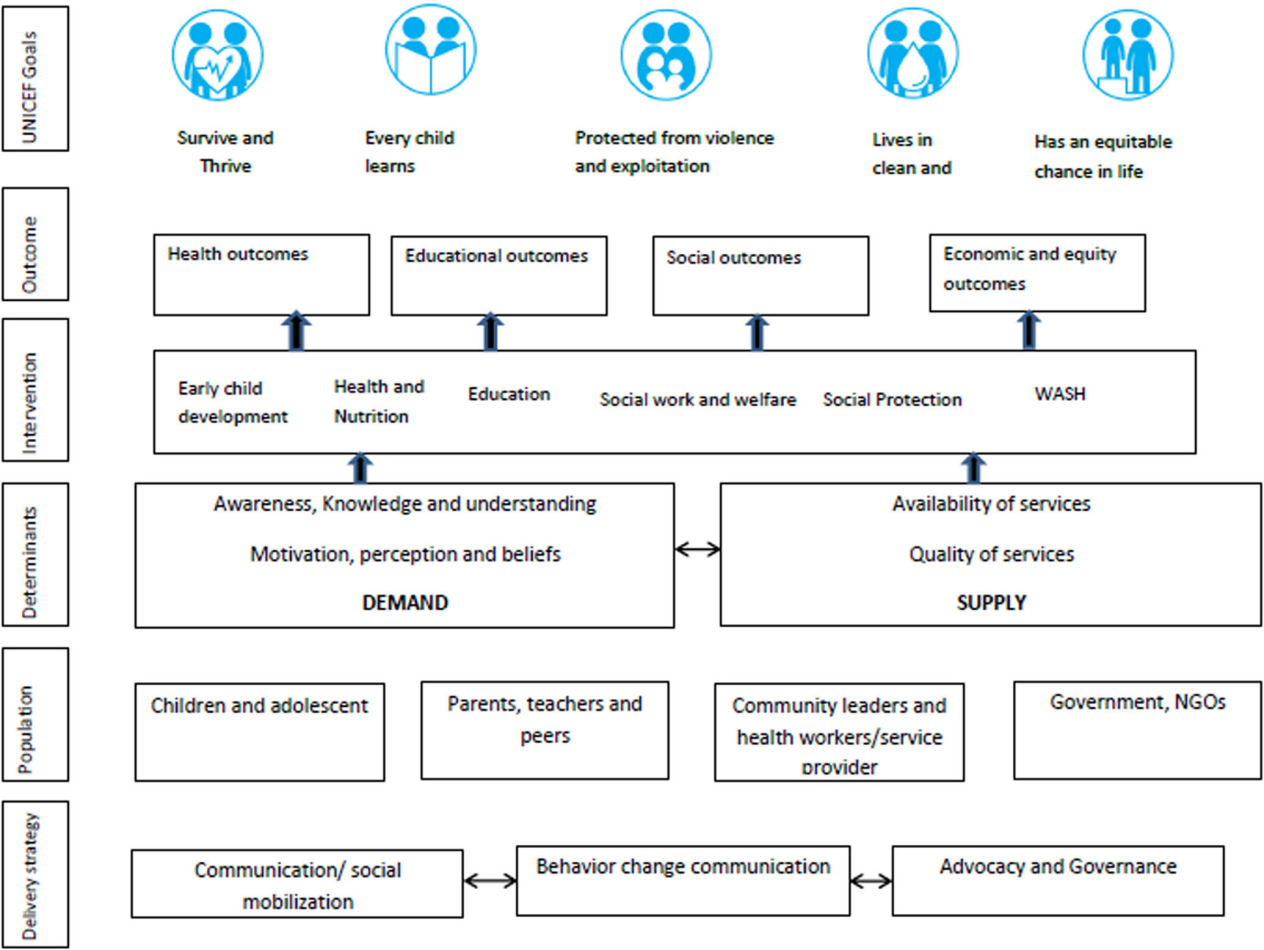
Conceptual framework for the child well‐being Megamap Source: Author's own design

Figure 1 below illustrates how child welfare interventions may help contribute to achieving UNICEF strategic goals outcomes.

### 1.7 Existing EGMs and/or relevant systematic reviews

Since this a Megamap and has a very broad scope, it will be first of its kind in this area. But there are related EGMs that will be included in the Megamap. Examples include

1. EGM on social, behaviour and community engagement intervention produced by World Health Organisation and International Initiative of Impact Evaluation (3ie) presents the evidence available on social, behavioural and community engagement interventions related to reproductive, maternal, newborn and child health programmes in LMICs.
2. EGM on primary and secondary education by 3ie that presents evidence on interventions designed to improve access to education and learning outcomes for primary and secondary school children in LMICs.
3. EGM on Intimate partner Violence highlights important gaps in the rigorous evidence base of intimate partner violence prevention programmes in LMICs.

There are number of focussed reviews related to specific child well‐being interventions such as

1. Systematic review by Bangpan, Dickson, Felix and Chiumento (2017) assess the impact of psychosocial interventions on mental health of children and adults in humanitarian emergencies.
2. Systematic review by Miller, Maguire and Macdonald (2011) assesses home‐based child development interventions for preschool children from socially disadvantaged families.
3. Another systematic review by Bright, Felix, Kuper and Polack (2017) measures effectiveness of interventions aimed at increasing access to health services for children aged 5 years and below in LMIC.

### 1.8 Objectives

The specific objectives are

1. Develop a clear taxonomy of interventions and outcomes related to the effectiveness of child well‐being interventions aimed at improving child well‐being in LMICs.
2. Map available systematic reviews and EGMs of the effectiveness of interventions aimed at improving child well‐being in LMICs with an overview provided in a summary report.
3. Provide database entries of included systematic reviews and EGMs which summarise the intervention, context, study design and main findings.

## 2 METHODOLOGY

### 2.1 Defining EGMs

This EGM is an effectiveness map in which the primary dimensions are the rows and columns of the map which are, respectively, intervention categories (and sub‐categories) and indicator domains (and sub‐domains). Secondary dimensions, such as country and target group will be included as filters.

### 2.2 Population

The primary population of interest for this Megamap is children under the age of 18 years as per the definition by United Nation Convention and includes children from LMICs. LMICs are defined by World Bank as low‐income economies ‐ those with a Gross National Income (GNI) less than $995; lower middle‐income economies – those with a GNI per capita between $996 and $3,895; and upper middle‐income economies – those with a GNI per capita between $3,896 and $12,055 (World Bank, 2018). Different child age ranges (0–1 month, 1 month–2 years, 2–6 years, 6–12 years and 12–18 years).

Population sub‐groups of interest include: orphans, children with disabilities, children belonging to ethnic minorities, child sex workers, malnourished children, child brides, isolated children/street child, children with HIV/AIDS and children in conflict and humanitarian settings.

### 2.3 Interventions

The included interventions cover all main strategies whose primary purpose is to improve child well‐being outcomes. So we do not include more general policies (e.g., macroeconomic policies) which will affect child well‐being, or more general social programmes (such as health or unemployment insurance). Many of the included interventions are directly targeted at children, for example, immunisation or education interventions. But that is not necessarily the case. For example, parenting programmes intended to modify parenting practice and a community‐based campaign against early marriage attempts to modify social norms – but in both cases with the end goal of improving child well‐being.

The seven intervention categories are

1. Early child development
2. Health and nutrition
3. Education
4. Social work and welfare
5. Social protection
6. Environment health including WASH
7. Governance

Table 1 lists the intervention sub‐categories under each of these headings. Intervention domains of the Megamap will be linked to UNICEF's five key goals proposed under the new Strategic Plan (2018–2021) and subsequent research briefs on findings from Megamap will be published for each of these UNICEF goals.

- Goal One: Every child survives and thrives
- Goal Two: Every child learns
- Goal Three: Every child is protected from violence and exploitation
- Goal Four: Every child lives in a safe and clean environment
- Goal Five: Every child has an equitable chance in life

**Table 1:** Intervention categories and sub‐categories

Intervention category	Intervention sub‐category
Early child development	Early childhood health intervention
	Early childhood nutritional interventions
	Early childhood education and parenting
	Women/maternal education and empowerment
Health and nutrition	Antenatal care, childbirth and post‐natal care by TBA/SBA
	Childhood immunisation
	Agricultural intervention/bio‐fortification
	Nutritional supplementation programme
	Management of severe acute malnutrition
	Community health interventions including CHWs
	Deworming
	Interventions for prevention and treatment of HIV/AIDs
	Prevention and management of childhood malaria
	Mass media campaigns on health education
	mHealth interventions for child health
	Maternal aid
	Mental health programme
Education	School voucher/reduced fees
	Decentralisation and local community participation
	School feeding programme and mid‐day meal
	School based health interventions
	Systemic renewal
	Alternative schooling/non‐formal education
	School sanitation and WASH
	Scholarship
	Teacher incentives
	Teacher training
	Remedial education
	Pedagogical approach
Social work and welfare	Birth registration
	Child‐trafficking preventions
	Intervention for child abuse
	Gender based violence programme
	Substance abuse prevention
	Child protection services
Social protection	Social insurance schemes
	Labour market insurance
	Social assistance interventions
Environmental health including WASH	Improved sanitation and water
	Hygiene education
	Prevention of outdoor and indoor air pollution
	Prevention of environmental tobacco smoke
	Prevention of exposure to toxins such as lead, mercury and pesticides
	Safe places to play
	Traffic calming
Governance	Child rights
	Legislative reforms
	Child protection regulation

### 2.4 Outcomes

The seven outcome categories are

1. Health
2. Healthy development
3. Learning and development
4. Risk factor reduction
5. Safety
6. Equity
7. Economic impact

Table 2 lists the outcome categories and sub‐categories. These are broad sub‐categories, and the recorded outcomes may be even positive or negative effects.

**Table 2:** Outcome categories and sub‐categories

Outcome category	Outcome sub‐category
Health	Mortality
	Morbidity
	Disability and childhood illness
	Immunisation coverage
	Mental health and psychosocial improvement
	Nutrition
Healthy development	Antenatal and postnatal care including breast‐feeding
	Cognitive development
	Utilisation of health services like immunisation, child care
	Gender roles/decision making
	Diet and physical activity
	Parent reported‐behaviour change
Learning and development	Enrolment
	Attendance
	Dropouts and truancy
	Learning and achievement
	Social skill development
	Quality of education
Risk factor reduction	Maternal smoking
	Contraceptive use
	Alcohol abuse/substance abuse
	Childhood injuries
	Hand washing
	Clean environment
Safety	Child abuse and neglect
	Homelessness
	Sexual (child trafficking)and physical violence in children
	Child rights
	Child marriage
	Child labour
	FGM prevalence
Equity	Equity
Economic impact	Cost‐benefit
	Cost‐effectiveness
	Formal savings

### 2.5 Criteria for including and excluding studies

#### 2.5.1 Types of study designs

The Megamap will include only systematic reviews and EGMs of effects of interventions. The key characteristics for a review to be included as a ‘systematic review’

1. A clearly stated set of objectives with pre‐defined eligibility criteria for studies.
2. An explicit, reproducible methodology.
3. A systematic search that attempts to identify studies that would meet the eligibility criteria.
4. A systematic presentation, and synthesis, of the characteristics and findings of the included studies.

Studies were not excluded based on the results of our critical appraisal of the included studies.

#### 2.5.2 Treatment of qualitative research

The map will not include qualitative research.

#### 2.5.3 Types of settings

Systematic reviews will be from LMICs. Systematic reviews that have a global focus will be excluded if their focus is predominantly on high‐income countries. Those which contain information on high‐, middle‐ or low‐income countries, but where the findings are clearly disaggregated by region or country and where the findings for low‐ or middle‐income regions or countries are presented separately will also be included.

EGMs that included studies from LMICs were included even if they had global focus.

#### 2.5.4 Status of studies

On‐going systematic reviews and EGMs will be included. Status of systematic reviews and EGMs will be a filter.

### 2.6 Search strategy and status of studies

The Megamap will be developed in three stages

- The first Stage 1 was a piloting stage for the framework which included search of relevant systematic reviews and EGM from 3ie databases. This search yielded 99 systematic reviews and 16 EGMs.
- Stage 2 based on a database search for a draft version of the map, which identified 302 systematic reviews and 16 EGMs
- Stage 3 for the Campbell map will include (a) search additional websites for grey literature, (b) consult experts and (c) screen submissions received in response to dissemination of the Stage 2 map.

The search will be as comprehensive as possible, using (but not limited to) relevant bibliographic databases and EGM databases, web‐based search engines, websites of specialist organisations, bibliographies of relevant reviews and targeted calls for evidence using professional networks or public calls for submission of articles. Additionally, reference lists of the included reviews will be reviewed and the authors contacted for information on other relevant sources.

### 2.7 Databases

1. International organisations
  ‐ UNICEF
  ‐ DFID (including Research for Development (R4D)
  ‐ UNFPA Evaluation Database
  ‐ WHO
2. EGM database
  ‐ 3ie EGM repository
  ‐ Swedish Agency For Health Technology Assessment and Assessment of Social Services
  ‐ Collaboration for Environmental Evidence
  ‐ Global Evidence Mapping Initiative
  ‐ Evidence based Synthesis Programme (Department of Veteran affairs)
  ‐ Cochrane
  ‐ Evidence based policing matrix
  ‐ EPPI Centre Evaluation Database of Education Research
3. Systematic review database
  ‐ Cochrane
  ‐ Campbell
  ‐ 3ie Systematic Review Database
  ‐ Research for Development
4. Academic databases
  ‐ World Bank eLibrary (Ebsco).
  ‐ The National Bureau of Economic Research (NBER)
  ‐ Social Science Research Network (SSRN)
  ‐ International Bibliography of Social Sciences (IBSS)
  ‐ Applied Social Sciences Index and Abstracts (ASSIA)
  ‐ Embase
  ‐ PsycINFO
  ‐ MEDLINE
  ‐ ERIC
5. Grey literature search/websites
  ‐ World Health Organisation
  ‐ World Bank
  ‐ UNICEF
  ‐ UNICEF Innocenti Research Centre
  ‐ UN Women
  ‐ UNESCO
  ‐ United Nations Population Fund
  ‐ UN Economic and Social Council
  ‐ CARE
  ‐ Save the Children
  ‐ African development bank
  ‐ Young Lives
  ‐ Association for the Development of Africa
  ‐ Médians Sans Frontières
  ‐ Action against Hunger
  ‐ World for World Organisation
  ‐ Project Concern
  ‐ One International
  ‐ World Vision
  ‐ Department for International Development
  ‐ World Food Programme
  ‐ Valid International
  ‐ Concern Worldwide
  ‐ Action Aid http
  ‐ CIFF
  ‐ International Red Cross
  ‐ WHO ICTRP
  ‐ Working Group on Early Childhood Development, Division for
  ‐ Social Policy & Development, Child Fund International
  ‐ GreyNet Internationa l
  ‐ Proquest Dissertations & Theses
  ‐ Opengrey
  ‐ Gates Foundation
  ‐ Clinton Foundation
  ‐ Abdul Latif Jameel Poverty Action Lab (J‐PAL
  ‐ Urban Youth Evidence Synthesis
  ‐ Innovations for Poverty Action (IPA) Database
  ‐ Child and Youth Finance International

Sample search is added as an Annexure A.

### 2.8 Screening and selection of studies

All titles and abstracts, and then full text, will be double screened, with a third party arbitrator in the event of disagreement.

### 2.9 Data extraction, coding and management

Coding will be done independently by two coders, with a third party arbitrator in the event of disagreement. The coding form is given in Annexure B. The coding form is very straightforward so coding is conducted in Excel.

### 2.10 Quality appraisal of systematic reviews

The quality of the included systematic reviews will be assessed using AMSTAR 2.

Critical appraisal will not be carried out for included EGMs.

### 2.11 Analysis and presentation

#### 2.11.1 Unit of analyses

Each entry in the map will either be a systematic review or and EGM. The accompanying EGM report will identify the number of systematic reviews and EGMs covered by the map in each sector.

#### 2.11.2 Presentation

The map will be generated using the EPPI Centre's Mapping Software.

In addition to intervention and outcomes, the following filters will be coded systematic reviews and Megamap:

(1) Population sub‐groups of interest include: orphans, children with disabilities, children belonging to ethnic minorities, child sex workers, malnourished children, child brides, isolated children/street child, children with HIV/AIDS and children in conflict and humanitarian settings, and different child age ranges. A study is coded by one of these filters if the sub group is the focus of the review.
(2) Region: East Asia & Pacific, Europe & Central Asia, Latin America & Caribbean, Middle East & North Africa, North America, South Asia, Sub‐Saharan Africa and conflict affected regions
(3) Systematic review quality: Based on AMSTAR 2 as high, medium and low
(4) Country classification by income level: low‐income <1,005, lower‐middle income 1,006–3,955 and upper‐middle income 3,956–12,235.

Filters 2 and 4 are applied if the included review or map's own inclusion criteria meant such studies were eligible. As mentioned above, the population sub‐group filters were applied when that was the focus of the review.

#### 2.11.3 Planned analysis

The EGM report shall provide tabulations or graphs of the number of systematic reviews and EGMs, with accompanying narrative description, by

- Intervention category and sub‐category
- Outcome domain and sub‐domain
- Table of ‘aggregate map’ of interventions and outcomes
- Region
- Year
- Study type and study quality

The map itself will show the number of studies in each cell which corresponds to an intervention sub‐ category and outcome sub‐domain. See Annexure C for snapshots of the draft map (the map itself may be found at https://cedilprogramme.org/wp‐content/uploads/2018/09/megamap_june252018.html).

### 2.12 Stakeholder engagement

The framework was developed through a consultative process. An advisory board was formed comprising of key experts in the area from UNICEF Innocenti, Ministry of Health and family welfare in India, African Child Forum etc.

1. Initial framework was developed based on various standard guidelines document of child well‐being by key organisations such as UNICEF, WHO, Save the Children.
2. The proposed framework was revised based on feedback by the advisory board and piloting of the framework was done using 3ie database search an screening.
3. The framework was then further revised in a mapping workshop in London where key stakeholders and experts in the area were invited. They reviewed the categories in an interactive exercise to fit the identified papers into the categories

The map will be discussed at various mapping workshop and advisory group meetings organised by UNICEF Innocenti and Campbell Collaboration.

## ROLES AND RESPONSIBILITIES

- *Content expertise*: K. A. who has experience regarding use of evidence across all of UNICEF's policy areas. H. W. who has published papers, including reviews and impact evaluations, on various aspects of child well‐being.
- *EGM methods expertise*: A. S. and H. W. have previous experience in systematic review methodology, including search, data collection, theory‐based synthesis, which mean they are proficient in carrying out the various processes in an EGM, such as search, eligibility screening, quality assessment and coding. They have undertaken an overview of approaches to mapping in a range of organisations. J. A. is experienced screener and has previously worked on Campbell Collaboration research projects.
- *Information retrieval expertise*: A. S. has training in designing and implementing search strategies.

## FUNDING

This EGM is partially supported by the UNICEF Innocenti Centre.

## CONFLICT OF INTERESTS

The authors declare that there are no conflict of interests.

## PRELIMINARY TIMEFRAME

Phase 1: Systematic reviews from 3ie database: completed

Phase 2: Full systematic review search: completed

Phase 3: *Grey literature search: Completed*

Protocol drafted: December 2018

Coding: October 2018

Draft report writing: June 2019

## PLANS FOR UPDATING THE EGM

Megamap will be updated annually and hence will again be updated in 2019. The EGM team are in discussions with the EPPI Centre, who are responsible for the mapping software, about possible real time updating through (a) automated searches with machine‐learning powered screening and (b) moderated submissions of suggested papers.

## ANNEXURE A

**Table:**

Search string/Key words (OVID platform)
Developing Country Free Text ‐ (developing OR less‐developed OR less* developed OR "under developed" OR underdeveloped OR under‐developed OR middle‐income OR "middle income" OR "low income" OR low‐income OR underserved OR "under served" OR deprived or poor*) adj3 (countr* OR nation OR population OR world OR state OR economy OR economies).mp ‐ ("third world" OR L&MIC OR L&MIC OR LAMIC OR LDC OR LIC OR lami countr* OR transitional countr*).mp ‐ (Africa OR "Sub‐Saharan Africa" OR "North Africa" OR "West Africa" OR "East Africa" OR Algeria OR Angola OR Benin OR Botswana OR Burkina Faso OR Burundi OR Cameroon OR "Cape Verde" OR "Central African Republic" OR Chad OR "Democratic Republic of the Congo" OR "Republic of the Congo" OR Congo OR "Cote d'Ivoire" OR "Ivory Coast" OR Djibouti OR Egypt OR "Equatorial Guinea" OR Eritrea OR Ethiopia OR Gabon OR Gambia OR Ghana OR Guinea OR Guinea‐Bissau OR Kenya OR Lesotho OR Liberia OR Libya OR Madagascar OR Malawi OR Mali OR Mauritania OR Morocco OR Mozambique OR Namibia OR Niger OR Nigeria OR Rwanda OR "Sao Tome" OR Principe OR Senegal OR "Sierra Leone" OR Somalia OR Somaliland OR "South Africa" OR "South Sudan" OR Sudan OR Swaziland OR Tanzania OR Togo OR Tunisia OR Uganda OR Zambia OR Zimbabwe).mp. ‐ ("South America" OR "Latin America" OR "Central America" OR Mexico OR Argentina OR Bolivia OR Brazil OR Chile OR Colombia OR Ecuador OR Guyana OR Paraguay OR Peru OR Suriname OR Uruguay OR Venezuela OR Belize OR "Costa Rica" OR "El Salvador" OR Guatemala OR Honduras OR Nicaragua OR Panama).mp. ‐ ("Middle East" OR "South‐East Asia" OR "Indian Ocean Island*" OR "South Asia" OR "Central Asia" OR Caucasus OR Afghanistan OR Azerbaijan OR Bangladesh OR Bhutan OR Burma OR Cambodia OR China OR Georgia OR India OR Iran OR Iraq OR Jordan OR Kazakhstan OR Korea OR "Kyrgyz Republic" OR Kyrgyzstan OR Lao OR Laos OR Lebanon OR Macao OR Mongolia OR Myanmar OR Nepal OR Oman OR Pakistan OR Russia OR "Russian Federation" OR "Saudi Arabia" OR Bahrain OR Indonesia OR Malaysia OR Philippines OR Sri Lanka OR Syria OR "Syrian Arab Republic" OR Tajikistan OR Thailand OR Timor‐Leste OR Timor OR Turkey OR Turkmenistan OR Uzbekistan OR Vietnam OR "West Bank" OR Gaza OR Yemen OR Comoros OR Maldives OR Mauritius OR Seychelles).mp. ‐ ("Pacific Islands" OR "American Samoa" OR Fiji OR Guam OR Kiribati OR "Marshall Islands" OR Micronesia OR New Caledonia OR "Northern Mariana Islands" OR Palau OR "Papua New Guinea" OR Samoa OR "Solomon Islands" OR Tonga OR Tuvalu OR Vanuatu).mp ‐ ("Eastern Europe" OR Balkans OR Albania OR Armenia OR Belarus OR Bosnia OR Herzegovina OR Bulgaria OR Croatia OR Cyprus OR "Czech Republic" OR Estonia OR Greece OR "Isle of Man" OR Kosovo OR Latvia OR Lithuania OR Macedonia OR Malta OR Moldova OR Montenegro OR Poland OR Portugal OR Romania OR Serbia OR "Slovak Republic" OR Slovakia OR Slovenia OR Ukraine).mp
Systematic review key words ‐ meta analysis/ ‐ ((systematic* adj2 review*) or "meta‐analy*" or "meta analy*")
Evidence map key words ‐ Evidence maps ‐ evidence adj3 map ‐ Evidence mapping ‐ Evaluation map ‐ Evaluation mapping ‐ Systematic map ‐ Systematic mapping ‐ Descriptive mapping ‐ Scientific uncertainties
Child well‐being key words ‐ [(child abuse* OR child welfare OR child well‐being OR child protect* service* OR foster care OR child* adj3 abus* OR schoolchild*, adolescen*, teen*)]
Target age group key words ‐ (young child* OR children OR pre‐schooler* OR pre‐schoolers*, kindergarten* OR kindergartener*, early child OR childhood, and early year OR years) ‐ ("adolescen*" OR juvenile OR minors OR youth OR "young adult" OR "young women" OR "young men" OR girl* OR boy* OR (school adj6 student*) OR teen* OR schoolgirl* OR schoolboy*)

## ANNEXURE B CODING

After screening, all systematic reviews and EGM will be coded for a wide array of information and populated into the map. The coded information includes bibliographic details for the study, the interventions from the framework that the study evaluates, the outcomes from the framework that the study measures and other relevant aspects. Coding of each study will be verified by a second researcher.

**Table:**

Coding variable	Example of information that may be recorded
Full references	Author, title, date of publication
Publication type	Academics, Journal, book, conference, thesis
Study Region	World Bank Region
Study Country	Name of the country
Status of study	On‐going/completed
Data Type	Qualitative/Quantitative
Child categories	Target group (e.g. child/adolescent)*
Intervention	Type of intervention/domain included like Early childhood intervention categories.
Population filters	Orphans/vulnerable children etc.
Outcome	Type of outcome assessed like safety, health etc.
Study Quality	Low, medium, high

## ANNEXURE D DEFINITION FOR INTERVENTIONS

**Table:**

Intervention Category	Intervention sub‐category	Category code	Examples
Early child development (ECD): *Encompasses physical, socio emotional, cognitive and linguistic development between 0‐8 years of age*	Early childhood Health intervention: This includes initiatives in health care, including health service provision, disease prevention, and health promotion to provide the continuum of maternal and child pre‐ and postnatal care.	ECD1	Health screening for pregnant women Maternal Immunization Birth spacing Cessation of smoking and substance misuse during pregnancy Support of mental health, New born screening Skilled attendants at birth, Childhood immunization, Prevention and integrated management of childhood illness Early identification through observation on child's behaviour Observation of parent‐child interaction Screening tests Identification and detection of risk factors, e.g. toxin exposure in utero, asphyxia, prematurity etc.
	Early childhood nutritional interventions: This includes initiatives to ensure that pregnant women, breastfeeding mothers, and young children are adequately nourished	ECD2	Promotion of adequate maternal nutrition Breast feeding promotion Supplementary feeding Dietary diversity Salt iodization Micro‐nutrient supplementation
	Early childhood education and Parenting: This includes interventions that provide opportunities for children to interact with responsive adults and actively learn with peers to prepare for primary school entry and also interventions directed on parent training.	ECD3	Pre‐schools/Pre‐primary/Kindergarten Day care/crèche Health based‐mother and child interventions Parenting education/programme Care institutions Child‐to‐child programmes
	Women/Maternal Education and empowerment: Interventions working with the mothers and families to change parents’ or caregivers’ knowledge, attitudes and behaviours or to encourage dialogue on health care services and decision making by women	ECD4	Participatory action groups on Gender norms Campaigns on maternal health education and girl child education Couples interventions Counselling (FP, ANC) for men and women, encourage men support Vocational training/life skill education Women support group on financial and gender issue
Health and Nutrition (HN): *A health intervention is an act performed for, with or on behalf of a person or population whose purpose is to assess, improve, maintain, promote or modify health, functioning or health conditions*.			
	Antenatal care, childbirth and post‐natal care by TBA/SBA: These interventions support and encourage women to adopt maternal health practices	HN1	receipt of HIV testing and PMTCT services ART adherence TT and intake of iron/folate supplements use ITNs Counselling on adequate nutrition and rest Awareness on exclusive breast‐feeding Family planning services
	Childhood Immunization: Promote and provide routine immunization/vaccination in infants/children	HN2	Training Supportive supervision Use of mass media Infrastructural development, e.g. provision of health facilities, provision of road to improve access to health facilities outreach; home visits; integration of vaccination with other services; IEC Parenting awareness on importance of immunization
	Agricultural intervention/bio‐fortification: Process to increase the density of vitamins and minerals in a crop through plant breeding, transgenic techniques, or agronomic practices.	HN3	
	Nutritional supplementation program: Interventions to promote supplementation of nutrients	HN4	Vitamin A supplementation from 6 months of age in Vitamin A deficient population Iron and folic acid supplementation Therapeutic zinc supplementation in children Iodization of salt/Iodine supplementation
	Management of severe acute malnutrition: Acutely malnourished children lack growth nutrients that are required to build new tissues. These nutrients aid weight gain after illness, repair damaged tissues and help replace the rapid turn‐over of cells (intestine and immune cells). Correct replenishment of nutrients like essential amino acids (protein), potassium, magnesium and zinc (among other minerals) is essential for recovery from malnutrition.	HN5	Targeted Nutrition supplementation Referral to hospital Recovery from diarrhoea Clinical Management
	Community health interventions including CHWs: Interventions in which communities direct the planning and implementation of intervention delivery	HN6	Observation of special days as Universal Children day on November 20 Health education classes for all sectors and cadre Participative sessions IEC through trained health workers/educational leaders/priests One‐time event‐delivery of key messages during youth days, school days, sports days etc. Performance monitoring of public health cadre (including pay for performance) Counselling sessions Health screening Building capacity Professional experience [continuing education/Training of Trainers (TOT)] ANC/PNC services Advocating community needs
	Deworming: Periodic treatment with anthelminthic (deworming) medicines, without previous individual diagnosis to preschool‐ and school‐aged children living in endemic areas.	HN7	
	Interventions for prevention and treatment of HIV/AIDs: Intervention related to HIV and other STIs among adolescents, including testing, incidence and prevalence.	HN8	Prevention of mother‐to‐child transmission (PMTCT) of HIV/AIDS, Paediatric HIV care, Nutritional Counselling, Free Integrated basic package of HIV care at all health facilities, Protection of human rights, Targeted interventions, HIV education and awareness, Condom social marketing program (CSMP) etc.
	Prevention and management of childhood malaria: Providing health and/or counselling services specific to childhood malaria in a community setting	HN9	Provision and promotion of use of insecticide treated nets (ITNs) for children Malarial prophylaxis in children
	Mass media campaigns on health education: Interventions employing mass media (for example, radio and television) to deliver health focused messages	HN10	Provide awareness on maternal and child health and increase demand of interventions
	mHealth interventions for child health: Interventions employing mHealth services or ICT approaches.	HN11	Examples include using particular websites such as Facebook SMS messages to provide health information. In some cases, the intervention itself is delivered on the internet.
	Maternal aid: Intervention that aim to provide subsidies that affect maternal and child health outcomes	HN12	
	Mental health program: Interventions to improve mental health of mother and child outcome	HN13	
Education (E): *Support needed to acquire the skills being taught by the educational system and should address functional skills, academic, cognitive, behavioral, and social skills that directly affect the child's ability to access education*			
	School voucher/reduced fees: Programmes providing allowances to cover all or some of the costs associated with education, including school fees, uniforms and books.	E1	
	Decentralization and local community participation: Interventions improving participation of community in effectively promoting education	E2	school‐based management community monitoring
	School feeding program and mid‐day meal	E3	
	School based health interventions: School health and nutrition programs that help children complete their education and develop health knowledge and lifelong positive behaviors	E4	Micro‐nutrient supplementation programmes Physical education and examination School‐based deworming programmes Zero tolerance policy‐ Enforcement of code of practice for teacher behaviour skills‐based education, including life skills, that addresses health, nutrition HIV/AIDS education and prevention awareness hygiene issues including menstrual hygiene and that promotes positive behaviour Counselling for bullying and abuse
	Systemic renewal: Systemic Renewal is “about continuous, critical inquiry into current practices, identifying innovations that might improve education, removing organizational barriers to that improvement, and providing a system structure that supports change”	E5	
	Alternative schooling/non‐formal education	E6	
	School sanitation and WASH: Interventions to ensure child friendly water supply, toilet and hand washing facilities in the schools and promote behavioral change by hygiene education	E7	
	Scholarship	E8	
	Teacher Incentives: Seek to improve the working conditions in schools so that teachers are motivated to come to work and improve their performance	E9	
	Teacher training: Interventions to improve the quality of instruction and offer more targeted tuition for children that are falling behind	E10	
	Remedial education: Also known as developmental education, basic skills education, compensatory education, preparatory education, and academic upgrading is assigned to assist students in order to achieve expected competencies in core academic skills such as literacy and numeracy.	E11	
	Pedagogical approach: Includes how the teacher interacts with students and the social and intellectual environment the teacher seeks to establish	E12	
Social work and welfare (SW): *development and provision of public or private social services to promote social justice amongst individuals and groups of individuals. While the term social welfare refers more generally to the well‐being of groups and individuals as well as the system of social service delivery, the term social work refers more specifically to the professional practice of delivering these social services*.			
	Birth registration	SW1	
	Child‐trafficking preventions	SW2	Strengthening police and judicial systems child law enforcement Registration of high risk groups Stringent laws on donations to beggars Awareness campaign Ensuring universal primary and secondary education programs
	Intervention for Child abuse: These interventions deal with prevention of child abuse.	SW3	Home visitation programs for victims Reduction of unintended pregnancies (ANC/PNC services) Reducing alcohol availability in high‐risk population Women Empowerment and Gender‐mainstreaming Changing cultural and social norms on violence against women Translating the convention on the rights of the Child into national
	Gender based violence program	SW4	
	Substance abuse prevention	SW5	Youth clubs School Based support groups Family support programs
	Child protection services: Child Protection Services deal with cases of abuse and severe neglect of children, and when a child seems to be suffering from, or is at risk of, significant harm	SW6	emergency protection of children who are suffering from actual/alleged child investigative assessment after receiving a referral long‐term follow‐up work with children and their families where abuse and/or neglect are verified formulate inter‐agency child protection guidelines and to work in liaison with the concerned agencies provide consultative services to agencies/organisations which come across cases of actual/suspected child abuse and/or neglect provide information and practical training to persons in contact with situations of child abuse and/or neglect. Re‐unification of families in conflict affected regions or refugees
Social Protection (SP) *is defined as the set of policies and programs designed to reduce poverty and vulnerability by promoting efficient labor markets, diminishing people's exposure to risks, and enhancing their capacity to protect themselves against hazards and interruption/loss of income*	Social insurance schemes: Microfinance, employability training, vocational training and savings programmes that aim to affect child welfare outcomes	SP1	Micro‐credit Microfinance Health financing
	Labor market interventions: Interventions covering work‐related injuries of employees, broadly affecting household income generation and child health	SP2	unemployment insurance, income support changes in labor legislation placement assistance job matching labor exchanges, Direct employment generation Training
	Social assistance interventions: Unconditional or conditional cash transfer programmes that aim to affect child welfare outcomes	SP3	Cash or In‐kind transfers such as food stamps and family allowances Voucher schemes User‐fee removal policy Temporary subsidies such as house subsidy in time of crisis
Environmental health including WASH (EH)	Improved sanitation and water	EH1	
	Hygiene education	EH2	
	Prevention of outdoor and indoor air pollution	EH3	
	Prevention of environmental tobacco smoke	EH4	
	Prevention of exposure to toxins such as lead, mercury and pesticides	EH5	
	Safe places to play	EH6	
	Traffic calming	EH7	
Governance (G): *“governance can be seen as the exercise of economic, political and administrative authority to manage a country's affairs at all levels. It comprises the mechanisms, processes and institutions, through which citizens and groups articulate their interests, exercise their legal rights, meet their obligations and mediate their differences.”*	Child rights: it provides a set of principles and standards covering children's entitlements to such essentials as education, healthcare and the right to be heard, as well as protection from abuses such as unjust treatment and exploitation. It places an obligation on states to ensure that all children within their jurisdiction (including non‐citizens such as refugees) enjoy these rights	G1	Educating children **on their rights** Supporting community‐based legal and paralegal services for children Child rights Monitoring **awareness** of legal drafting committees on the importance of children's rights and the provisions of the UNCRC. Provide technical assistance and training to law enforcement officials, judges, parliamentarians and others concerned with implementations Advocate for legislative costing and appropriate resource allocation ensuring the availability of financial and other assistance for equitable access for children and families to judicial system Using available complaint mechanism of child right commissions in countries Support **child‐led media initiatives** building the **capacity of media organisations on children's rights**
	Legislative reforms: develop and adopt national legislation to implement child protection laws.	G2	family law juvenile justice laws about education against child labour laws to protect children from sexual exploitation Gender quality Non‐discrimination
	Child protection regulation: Measures that protect access to resources, promote employment, and support the childcare role.	G3	national budget allocation and expenditure for maternal and child services. Adopting national legislation for birth registration abolishing child and bonded labour enforcing minimum wage rates, providing paid maternity leave ensuring that health and safety standards are met. Inter‐agency collaboration in areas of conflicts to address child protection needs
